# Genetic diversity analysis of Thai indigenous pig population using microsatellite markers

**DOI:** 10.5713/ajas.18.0832

**Published:** 2019-03-07

**Authors:** Rangsun Charoensook, Kesinee Gatphayak, Bertram Brenig, Christoph Knorr

**Affiliations:** 1 Division of Animal Science and Feed Technology, Department of Agricultural Sciences, Faculty of Agriculture Natural Resources and Environment, Naresuan University, Phitsanulok 65000, Thailand; 2 Department of Animal and Aquatic Science, Faculty of Agriculture, Chiang Mai University, 50200 Chiang Mai, Thailand; 3 Division of Molecular Biology of Livestock and Molecular Diagnostics, Faculty of Agricultural Sciences, Georg-August University of Göttingen, Göttingen 37077, Germany; 4 Division of Livestock Biotechnology and Reproduction, Faculty of Agricultural Sciences, Georg-August University of Göttingen, Göttingen 37077, Germany

**Keywords:** Genetic Diversity, Microsatellite Markers, Pigs, Phylogenetic Analysis, Thailand

## Abstract

**Objective:**

European pigs have been imported to improve the economically important traits of Thai pigs by crossbreeding and was finally completely replaced. Currently Thai indigenous pigs are particularly kept in a small population. Therefore, indigenous pigs risk losing their genetic diversity and identity. Thus, this study was conducted to perform large-scale genetic diversity and phylogenetic analyses on the many pig breeds available in Thailand.

**Methods:**

Genetic diversity and phylogenetics analyses of 222 pigs belonging to Thai native pigs (TNP), Thai wild boars (TWB), European commercial pigs, commercial crossbred pigs, and Chinese indigenous pigs were investigated by genotyping using 26 microsatellite markers.

**Results:**

The results showed that Thai pig populations had a high genetic diversity with mean total and effective (N_e_) number of alleles of 14.59 and 3.71, respectively, and expected heterozygosity (H_e_) across loci (0.710). The polymorphic information content per locus ranged between 0.651 and 0.914 leading to an average value above all loci of 0.789, and private alleles were found in six populations. The higher H_e_ compared to observed heterozygosity (H_o_) in TNP, TWB, and the commercial pigs indicated some inbreeding within a population. The Nei’s genetic distance, mean *F*_ST_ estimates, neighbour-joining tree of populations and individual, as well as multidimensional analysis indicated close genetic relationship between Thai indigenous pigs and some Chinese pigs, and they are distinctly different from European pigs.

**Conclusion:**

Our study reveals a close genetic relationship between TNP and Chinese pigs. The genetic introgression from European breeds is found in some TNP populations, and signs of genetic erosion are shown. Private alleles found in this study should be taken into consideration for the breeding program. The genetic information from this study will be a benefit for both conservation and utilization of Thai pig genetic resources.

## INTRODUCTION

Native pigs and wild boars are indigenous to Thailand whereas European pigs (e.g. Duroc [DR], Large White [LW], and Pietrain [PT]) were first imported in 1950s [1]. The domestic animal diversity information system (DAD-IS; http://www.dad.fao.org) of the Food and Agriculture Organization (FAO) has listed Thai native pigs (TNP, *Sus scrofa*) into four breeds, namely Raad (or Kadon), Puang, Hailum and Kwai, according to their physical appearance and the region where they are predominant [2,3]. However, these breeds have been classified for some 40 to 50 years, so it is nowadays difficult to determine any unique characteristics which are specific for each pig breed. Moreover, since 1980s native pigs have been increasingly mated with European pigs to improve their economically important traits which eventually led to the development of Thai pork industry. Finally, most native pigs were gradually replaced by pure European commercial breeds and have become even less suited for the breeding company’s needs, so the number of indigenous pigs in Thailand has steadily decreased over the years [3].

A survey in 1994 [2] reported that less than 500 herdbook native sows and less than 10 nucleus herds were registered. Thai indigenous pigs are nowadays particularly kept in small populations by smallholders in the Northern and North-Eastern provinces of Thailand for reasons of tradition and of religion [3]. However, small pig populations without any scrutinized breeding programs risk to losing their genetic diversity and identity, and becoming extinct. The conservation and utilization of indigenous genetic resources are important to animal sciences. For successful conservation, a detailed knowledge of a breed’s phenotypic trait and genetic background are thus very important to study their genetic diversity [4]. Microsatellite markers have been proven to be an extremely powerful tool for analyzing genetic diversity and phylogenetic relationships in animals [e.g. 5–9]. Although numerous reports on foreign pig breeds are available, Thai pig genetics resources are still mostly unknown due to a little study on them [8–10].

Therefore, we performed large-scale genetic diversity and phylogenetic analyses using genomic data on many pig breeds available in Thailand (indigenous, crossbred, commercial exotic breeds, etc.) and Chinese pigs.

## MATERIALS AND METHODS

Research protocols and experimental procedures were approved by the guidelines stated in the Guide for Care and Use of Agricultural Animals in Agricultural Research and Teaching [11].

### Resource populations

Samples of 72 TNPs and 11 Thai wild boars (TWB) were used. Blood, ear clips or hair samples were collected from twelve localities/amphurs of five Northern provinces, namely Mae Hongson (MH), Chiang Mai, Chiang Rai (CR), Uttaradit and Nan. The TNPs consisted of five native pig populations (Table 1). TWBs were considered as one population, as they remained isolated in the wilderness of Chiang Mai and Nan provinces. Three populations of purebred European pigs and two crossbreds between European and Thai pigs were included as well. Collectively, these 11 pig populations were considered as Thai pigs in this study. Additionally, genotypic information on six Chinese breeds were included from our DNA repository in University of Goettingen to compare Thai pigs genetic diversity in reference to Asian pig genetic resources. The Chinese pigs were selected based on different geographical distributions and ecological types reflected by different phenotypic and morphological characters. The Chinese pigs were as follows: Jiangquhai (JQH; lower Yangtze river basin type), Luchuan (LC; South China type), Minpig (MZ; North China type), Rongchang (RC; Southwest China type), Yushanhei (YJ; Central China type), and Tibetan (TI; plateau type) with 10 animals each [12]. Finally, the study analyzed microsatellite markers from a total of 222 pigs.

### Molecular genetics analyses

Genomic DNA was extracted from whole blood (9 mL vials containing ethylenediaminetetraacetic acid) and ear clips by a modified salting out method according to Sambrook et al [13] and Miller et al [14] or from hair roots using the QIAamp DNA mini kit (Qiagen, Germany).

A panel of 26 microsatellites markers covered all porcine chromosomes including the sex chromosomes was analyzed (Table 2, Supplementary Table S1). Primers were fluorescently labelled with dyes FAM or HEX at the 5′-end. Polymerase chain reaction (PCR) assays were performed using 50 to 100 ng of genomic DNA, 2.5 mM MgCl_2_, 0.2 mM of each dNTP, 0.4 μM of each primer, and 0.5 units of *Taq* polymerase (Qiagen, Hilden, Germany) in 1× PCR buffer as recommended by the manufacturer in a final volume of 25 μL. The PCR profile consisted of 35 cycles at 94°C for 30 s, the specific annealing temperature for 30 s (see Supplementary Table S1), and an extension period of 30 s at 72°C with an initial denaturation for 2 min at 94°C and a final extension at 72°C for 5 min. PCR reactions were performed on a Biometra T-Gradient thermocycler (Biometra, Goettingen, Germany). To check fragment integrity, PCR products were loaded on 2% agarose gels.

### Genotyping

For genotyping of samples, the size separation and fragment analysis were performed on an ABI PRISM 3100 DNA analyzer (ABI, Weiterstadt, Germany), using GENESCAN-500ROX as internal size standard according to the manufacturer’s specifications. Evaluation of microsatellites and size determination of alleles were done with appropriate ABI-software GENESCAN and Gentoyper software (Applied Biosystems, Waltham, MA, USA), respectively.

### Data analyses

Genetic diversity parameters were estimated for each microsatellite locus and across all loci for each population by the mean number of alleles (MNA), effective number of alleles (N_e_), observed heterozygosity (H_o_), expected heterozygosity (H_e_), and possible deviations from the Hardy-Weinberg equilibrium using GENETIX 4.03 [15] and POPGENE 1.31 [16]. The polymorphism information content (PIC) per locus [17] was calculated by CERVUS 3.0.3 [18]. The inbreeding coefficient of an individual relative to the total population (*F*_IT_), effect of subpopulations compared with the total populations (*F*_ST_), and inbreeding coefficient of an individual relative to the subpopulation (*F*_IS_) and values for each breed were calculated using the FSTAT 2.9.3 [19].

Nei’s genetic distance was computed between populations using POPGENE 1.31 [16]. This matrix of genetic distances was used to construct a phylogenetic tree with 1,000 replicates to obtain the corresponding bootstrapping values and to assess the robustness of the dendrogram topology by the neighbour-joining (NJ) method [20] using the PHYLIP package [21]. The dendrogram was depicted using the Tree view software package 1.6.6 [22]. Moreover, the software program GenAlEx 6 [23] was used to conduct two-dimensional data (MDS-2D) based on pair-wise proportion of different alleles, to visualize their similarity or dissimilarity among 17 pig populations. Finally, genetic distances among 222 individuals were estimated as the proportion of shared alleles and represented by a NJ tree using the molecular evolutionary genetics software version 4.0 [24].

## RESULTS AND DISCUSSION

The ISAG/FAO Standing Committee for biodiversity has recommended a panel of 27 pre-selected porcine microsatellites [25] However, in this present study 24 of the recommended 27 microsatellites were used, because of three markers (S0178, S0228, and SW24) have presented unreliable chromatogramms for fragment analysis. Thus, those markers were replaced by markers S0120 and SW1031 to still cover all porcine chromosomes including the sex chromosomes.

### Microsatellite polymorphisms

In total, 367 alleles were observed at the 26 loci. The total number of alleles per locus varied from 7 (SW951) to 29 (CGA) with a global mean of 14.59 alleles per locus (Table 2). All microsatellites revealed high degrees of polymorphism and allelic diversity. MNA per marker ranged between 3.82 (SW951) and 10.64 (CGA) with an overall mean of 5.65. N_e_ ranged between 2.62 (S0218) and 7.15 (CGA) with a pooled mean of 3.71. For nine of the 26 loci private alleles were described. The highest number of specific alleles per marker was visible for SW122 (three). Only for three alleles, frequencies of 10% or higher were observed (allele 229 at S0227; allele 107 at SW122, and allele 255 at S0068). Genotyping of further individuals should help to verify at the population level which of these alleles are at low frequency or not at all present in the respective pig sources. The highest frequency of specific alleles per population was observed for TWB and pigs collected in the Uttaradit province. Our data support Thuy et al [26] who also reported new alleles per locus present in the indigenous breeds in their comparative study of Vietnamese and European pigs.

The PIC per locus was highest (0.914) for CGA and lowest (0.651) for SW951 leading to an average value above all loci of 0.789, which is superior to the one of 0.755 of the Thai pigs investigated by 8 and the one of 0.685 reported for Portuguese breeds [6]. The overall H_o_ for our Thai pigs was 0.679 and the H_e_ was 0.710. Vincente [6] reported lower values of 0.621 H_e_ respectively and 0.667 for H_o_ respectively. H_e_ For Mexican Creole pigs’ values of 0.46±0.04 (H_o_) and of 0.72±0.04 for H_e_ were described [27]. Fabuel et al [28] introduced a H_o_ of 0.576 and a H_e_ of 0.697 calculated for their Iberian pigs. Chaiwatanasin et al [8] documented a mean H_o_ of 0.534 and a mean H_e_ of 0.793. Wright’s *F*-statistic estimates were calculated for each locus (Table 2).

The divergence between H_e_ and H_o_ for all individuals is expressed as the total inbreeding estimate (*F*_IT_), with a mean of 0.169 (vary between 0.013 for S0226 and 0.583 for S0386). This value is lower compared to Chinese pigs [29,30] and Portuguese pigs [6]. Our observed *F*_IS_ of 0.007 was found to be lower than the those of others (0.274) [30], 0.067 [6], and 0.21 [29]. However, only S0386 (0.518) and S0218 (0.377) were found to be higher than the published data [29,30]. The excess of *F*_IS_ or the reduction between H_o_ and H_e_ of microsatellite markers in this study might be caused by null alleles or population subdivisions [31].

The multi-locus *F*_ST_ which expresses the population differentiation averages 0.162. However, the estimated *F*_ST_, with respect to a single locus, showed considerable variations in this study i.e., 0.07 in CGA and 0.249 in S0218. This illustrates that only 16% of genetic variance in our studied populations was explainable by population structures. Thus, in other terms, most genetic variation or diversity was within populations. The comparison of *F*_ST_ between our study and the previously published study by Yang et al [30], Li et al [29] and Vicente et al [6] demonstrate that the values were in the same range (0.077 to 0.022).

### Genetic diversity in Thai pig populations

Table 3 depicts the assessment of genetic diversity among Thai pig populations. Over all, mean MNA and N_e_ were high in Thai native pigs (TNP) and TWB. Only pigs from MH revealed fewer superior values where both MNA and Ne were comparatively lower than some of the commercial pigs and crossbreds. A higher allelic diversity in indigenous breeds is known [26] and probably the consequence of the lack of planned mating programmes. The higher H_e_ compared to H_o_ in TNP, TWB, and the commercial pigs indicated some inbreeding within population but opposed to the crossbred pigs that revealed a surplus of heterozygous animals (H_o_>H_e_). A high heterozygosity must be attributed to heterosis, and at the same time to a marginal degree of inbreeding effects. In their earlier study, Chaiwatanasin et al [8] described estimates for H_e_ and H_o_ in TNP and commercial Thai pigs represented by the breeds LW, PT, and Spotted Large White (SLW). Their report stated small H_o_ differences between populations only. It was, however, unexpected that their estimates for H_o_ in LW and SLW but PT were higher as compared to TNP. The highest H_e_ was computed for TNP. Pigs in [8] revealed a larger ratio of H_e_ and H_o_ (0.534 and 0.793) due to smaller H_o_ compared to pigs in this study. However, we could not verify our results with them as their animals’ detailed origin information was unavailable [8].

Most inbreeding coefficients (*F*) in this study were either zero (crossbreds) or small (commercial breeds) (Table 3). No inbreeding or excess heterozygosity in crossbreds could be largely due to the presence of heterosis. However, the low or negligible *F* in commercial pigs could be caused by the sophistication of the breeding programs [3,8,9]. However, some inbreeding signs were found in pigs from southern part of Chiang Mai Province (SCM; 0.139), Chiang Rai Province (CR; 0.105) and the TWB (0.100). It is assumed that inbreeding between closer relatives might have occurred. For example, in a non-inbred population, a *F* of at least 0.125 is expected to occur if either grand-father/grand-daughter (grand-mother/grand-son), alternatively half-brother/half-sister or uncle/niece (aunt/nephew) are mated. We cannot rule these scenarios out as animals of these populations were kept in small villages for generations (SCM and CR) or were caught and kept in captivity as it was in the case of the TWB.

### Genetic distances and phylogenetic relationships between pig populations

Genetic distances for the Thai and Chinese pig populations (JQH, LC, MZ, YJ, TI, and RC; Table 1) were assessed according to Nei [32] and by mean *F*_ST_ estimates (significance was tested using the permutation test). Pair-wise comparisons for all pig populations are shown in Table 4. Nei’s estimates indicated a higher than expected genetic distance between TWB and TNPs (from 0.352 to 0.606). It was interesting that the genetic distance among TWB and DR and PT (0.579 and 0.567) was even lower than the one to MH (0.606). However, expectedly, those of crossbreds estimates were somewhere in between the ‘founder’ breeds. Larger genetic distances (≥0.439, expect for TI to RC) were also found between those of Chinese breeds, especially between LC and JQH (0.741). We also observed greater genetic distances between Thai and Chinese pigs. The closest relationships were, however, estimated between the Chinese breeds (TI and RC), and between TNP populations (SCM and CR). For TWB, its closest neighbour among the Chinese breeds was TI (*F*_ST_: 0.082). This situation might be possible that TI pigs have domesticated from wild boars in the near past with least breeding and selection by human.

The overall *F*_ST_ of 0.162 indicates significant population subdivision in the studied Thai pig breeds. Laval et al [33] reported higher estimates for European breeds (*F*_ST_ = 0.27). Lower values *F*_ST_ were also reported in Chinese breeds (0.077) [30]. Our pair-wise *F*_ST_ estimates ranged from 0.037 (between CR and SCM) to 0.235 (between LW and LC). These estimated were within the ranges from various early reports such as reports using microsatellite markers in European breeds (0.11 to 0.27) [6,33,34] or reports in Chinese and Korean breeds (0.18 to 0.26) [5,29,35]. The wider range of *F*_ST_ estimates between of TNP and European breeds also coincided with those reported by Chaiwatanasin et al [8]. On the contrary to that TWB are genetically more distant to European pigs. Note that the existing disagreements among reports are probably due to distinctive marker sets and quality of animal samples i.e. randomly sampled [5] or not.

**Figure 1:**
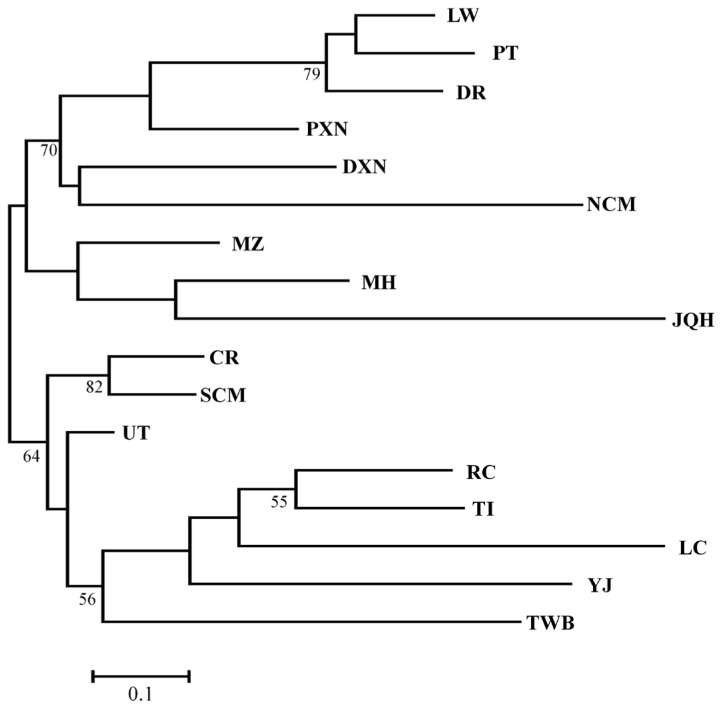
Representation of neighbour-joining Nei’s (1972) standard genetic distance among Thai and Chinese pig populations, based on 1,000 replicates (numbers in nodes are percentage bootstrap values). LW, Large White; PT, Pietrain; DR, Duroc; PXN, Pietrain×native crossbred pig; DXN, Duroc×native crossbred pig; NCM, Northern part of Chiang Mai; MZ, Min; MH, Mae Hongson; JQH, Jiangquhai; CR, Chiang Rai; SCM, Southern part of Chiang Mai; UT, Uttaradit; RC, Rongshang; TI, Tibetan; LC, Luchuan; YJ, Yushanhei; TWB, Thai wild boars.

A phylogenetic tree consisting of the 17 populations was reconstructed based on Nei’s genetic distances (Table 1, Figure 1) [36], and the tree could be distinguished into two distinct clusters. The first cluster consisted of three commercial breeds, all crossbred pigs, two TNPs (Northern part of Chiang Mai Province [NCM] and MH) and two Chinese populations (MZ and JQH). The additional Chinese breeds arranged with TWB and three TNPs (CR, SCM, and UT) and formed the second cluster. The phylogenetic tree infers a close relationship between Thai native and Chinese pigs. At the same time both populations are distinctly different from European lineages.

**Figure 2:**
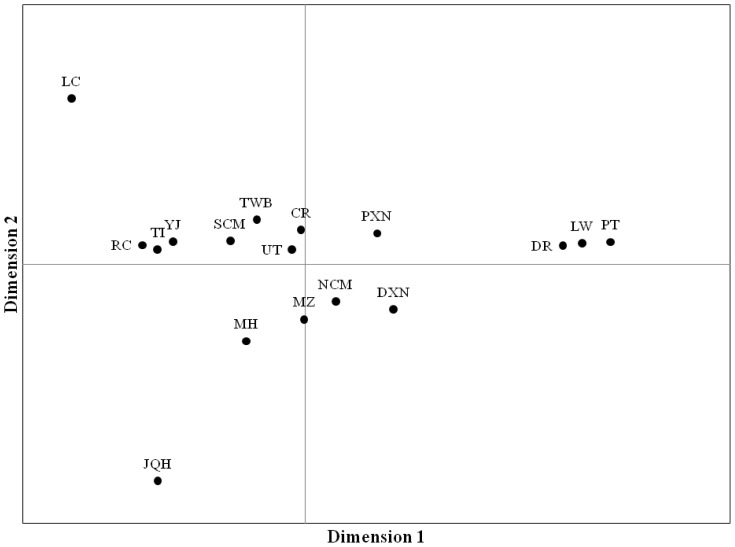
Multi-dimensional scaling (MDS) in a two-dimensional area based on pair-wise proportion of different alleles (*F*_ST_) among 17 pig populations. Axis1 = 43.21 %, Axis2 = 25.22%. See Table 1 for population abbreviations. LC, Luchuan; RC, Rongshang; TI, Tibetan; YJ, Yushanhei; SCM, Southern part of Chiang Mai; TWB, Thai wild boars; CR, Chiang Rai; UT, Uttaradit; PXN, Pietrain×native crossbred pig; DR, Duroc; LW, Large White; PT, Pietrain; NCM, Northern part of Chiang Mai; DXN, Duroc×native crossbred pig; MZ, Min; MH, Mae Hongson; JQH, Jiangquhai.

Multidimensional scaling (MDS) in a two-dimensional area (Figure 2) was further computed to display genetic similarities among populations based on the pair-wise proportion of different alleles (*F*_ST_). The MDS showed that European pigs separated clearly from each other, the crossbreds and the NCM populations formed a second group, which separated Asian pigs from European pigs. This indicated that crossbreeding events with individuals on commercial farms might have occurred in NCM population.

**Figure 3:**
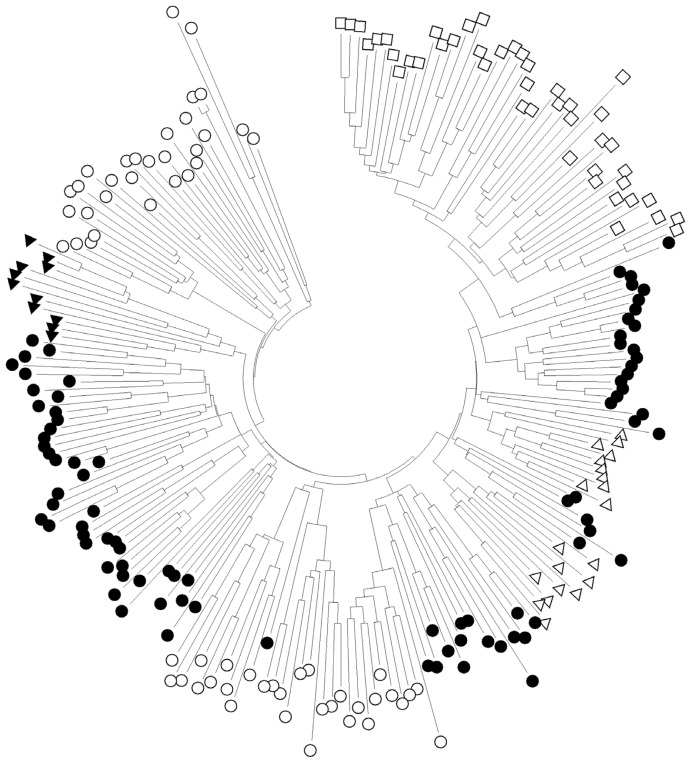
Neighbour-joining tree based on the proportion of shared alleles of Thai native pigs (black circle), Thai wild boars (black triangle), Commercial crossbred pigs (white triangle), Commercial pigs (white square), and Chinese pigs (white circle).

To analyse the genetic admixture in each pig population, NJ tree of individuals was constructed based on Nei’s unbiased genetic distance [36] of the shared allele proportions (Figure 3). The genetic differentiation among different pig breeds or populations was probably due to selection, genetic drift, and local inbreeding effects. On the other hand, the close genetic relationship between some TNPs and the crossbreds could be a direct effect of a genetic introgression from European pigs. In 1957, the Department of Livestock Development at the Ministry of Agriculture, under the guidance of FAO, started to import European pig breeds into Thailand and has promoted for the production of crossbred pigs for sale in local areas [2].

### Implications of Thai pig genetic resources

The term “livestock genetic resources” has included all livestock species, breeds and strains that are of economic, scientific and cultural interest to humankind in terms of sustainable food and agricultural production [3]. Accordingly, TNPs are well adapted to hot and humid climates, tolerate to low quality of feed stuff, and probably are better resistant to internal parasites and viral diseases [2]. Moreover, some study has reported that 24 TNPs in Lamphun province remained healthy with complete protection and no symptoms while the foot and mouth virus attacked all cattle of the village. This showed that TNPs could resist the virus which was confirmed by a low antibody titre (less than 40%) [37]. Thus, it seems likely that Thai indigenous pigs might be a useful genetic resource.

The primary focus of our study was to evaluate the genetic background of pigs in Northern Thailand that are the main genetic resource of native pigs in this country. Our previous study had described specific mtDNA signatures for TNPs and TWBs [10]. In this present study, the private microsatellite alleles which are recommended to be conserved in populations are found here for UT (S0155, SW240, S0002), NCM (S0002, S0225), TWB (S0227, SW122), CR (SW911), SCM (S0068), and DR (SW1031) and will provide additional information to genetically describe the uniqueness of TNPs and TWB. It should therefore be a benefit for both conservation purposes as well as to utilizing them for a sustainable pig production. However, since the knowledge of indigenous pig genetics is still limited, a further collaboration among the agencies within and between countries is required [3].

In conclusion, microsatellite loci analyses indicated close genetic relationship between Thai indigenous and Chinese pigs. NCM pig population showed a genetic introgression from European breeds, and some Thai indigenous pigs (SCM, CR, TWB) showed signs of genetic erosion. It could be assumed that genetic diversity of Thai indigenous pigs is a product of different husbandry systems and lack of suitable breeding programs. Urgent measures of conservation and sustainable management of their gene pool must be undertaken, and private alleles which found in this study should be taken into consideration for the breeding program.

## Supplementary Data



## Figures and Tables

**Table 1:** Animals and sampling information

Population or breed samples	Sampling location	N	Type of samples
Thai native pigs (TNP)			
Native pig I (MH)	Mae Hongson Province	6	Ear clip
Native pig II (SCM)	Southern part of Chiang Mai Province	29	Blood, ear clip, hair
Native pig III (NCM)	Northern part of Chiang Mai Province	20	Blood, ear clip
Native pig IV (CR)	Chiang Rai Province	11	Blood, ear clip
Native pig V (UT)	Uttaradit Province	21	Blood, ear clip
Thai wild boars			
Wild boar (TWB)	Chiang Mai & Nan Provinces	11	Blood, ear clip
Commercial pigs			
Duroc (DR)	Chiang Mai Province	22	Blood
Pietrain (PT)	Chiang Mai Province	10	Blood
Large White (LW)	Chiang Mai Province	12	Blood
Commercial crossbred pigs			
Duroc×native crossbred pig (DXN)	Chiang Mai Province	10	Blood
Pietrain×native crossbred pig (PXN)	Chiang Mai Province	10	Blood
Chineses indigenous pigs			
Jiangquhai (JQH)	Jiangsu Province	10	Blood
Luchuan (LC)	Guangxi Province	10	Blood
Min (MZ)	Liaoning, Jilin & Heilongjiang Province	10	Blood
Yushanhei (YJ)	North-Eastern Jiangxi Province	10	Blood
Tibetan (TI)	Tibet & Yunan Province	10	Blood
Rongshang (RC)	Western Sichuan Province	10	Blood
Total		222	

N, number of samples.

**Table 2:** Characterization of the 26 microsatellites in Thai pigs

Locus	SSC	TNA	MNA	Ne	PIC	Ho	He	FIT	FST	FIS	HWE	Private allele; bp (animal and frequency)
*S0155*	1	10	5.73	3.58	0.835	0.736	0.733	0.139	0.179	−0.049	NS	143 (UT 0.05)
*CGA*	1	29	10.64	7.15	0.914	0.761	0.889	0.171	0.072	0.107	**	
*S0226*	2	11	4.45	3.04	0.761	0.785	0.669	0.013	0.193	−0.224	*	
*SW240*	2	14	5.36	3.60	0.828	0.741	0.708	0.127	0.199	−0.091	**	120 (UT 0.07)
*S0002*	3	27	7.91	5.25	0.896	0.839	0.794	0.068	0.155	−0.103	NS	204 (UT 0.05); 252 (NCM 0.05)
*SW72*	3	10	5.36	2.95	0.697	0.617	0.656	0.141	0.124	0.020	NS	
*S0227*	4	9	4.09	2.85	0.707	0.683	0.648	0.068	0.154	−0.102	NS	229 (TWB 0.14)
*IGF-1*	5	10	5.36	3.84	0.795	0.765	0.738	0.060	0.131	−0.082	NS	
*S0005*	5	19	7.00	4.73	0.861	0.671	0.810	0.241	0.123	0.134	ND	
*SW122*	6	13	5.64	3.49	0.845	0.728	0.712	0.156	0.208	−0.066	NS	99 (TWB 0.09); 107 (TWB 0.23); 109 (TWB 0.09)
*S0101*	7	11	4.73	2.89	0.778	0.633	0.654	0.234	0.241	−0.009	**	
*SW632*	7	11	6.00	3.58	0.823	0.635	0.732	0.248	0.168	0.095	**	
*S0225*	8	13	5.09	3.34	0.767	0.778	0.700	0.017	0.152	−0.160	NS	173 (NCM 0.05)
*SW911*	9	10	6.00	3.87	0.818	0.734	0.729	0.112	0.152	−0.047	*	144 (CR 0.09)
*SW951*	10	7	3.82	2.69	0.651	0.554	0.645	0.210	0.119	0.103	*	
*S0386*	11	9	4.18	2.84	0.663	0.293	0.635	0.583	0.135	0.518	**	
*S0090*	12	10	5.18	3.48	0.814	0.785	0.701	0.062	0.196	−0.167	NS	
*S0068*	13	17	7.45	5.08	0.884	0.878	0.802	0.018	0.141	−0.142	NS	255 (SCM 0.16)
*S0215*	13	13	4.45	3.07	0.737	0.553	0.580	0.251	0.246	0.007	**	
*SW857*	14	11	5.73	4.11	0.852	0.633	0.755	0.269	0.163	0.127	NS	
*S0355*	15	12	5.82	3.80	0.815	0.685	0.697	0.179	0.199	−0.025	NS	
*SW936*	15	18	6.09	4.12	0.836	0.840	0.764	0.029	0.153	−0.146	**	
*S0026*	16	9	5.09	3.35	0.762	0.731	0.715	0.083	0.140	−0.067	NS	
*SW1031*	17	11	4.82	2.99	0.696	0.498	0.659	0.319	0.137	0.211	**	116 (DR 0.07)
*S0120*	18	15	7.00	4.34	0.797	0.773	0.784	0.062	0.089	−0.029	NS	
*S0218*	X	11	4.09	2.62	0.685	0.335	0.562	0.532	0.249	0.377	**	
Mean		14.59	5.65	3.71	0.789	0.679	0.710	0.169	0.162	0.007		

SSC, *Sus scrofa chromosome*; TNA, total number of alleles per locus; MNA, mean number of alleles per locus; N_e_, effective number of alleles per locus; PIC, polymorphism information content, H_o_ and H_e_, the observed and unbiased expected heterozygosity; F_IT_, inbreeding coefficient of an individual relative to the total population; F_ST_, effect of subpopulations compared with the total populations; F_IS_, inbreeding coefficient of an individual relative to the subpopulation; HWE, Hardy-Weinberg equilibrium; NS, no significant difference; ND, not done.

* p<0.05;

** p<0.01.

**Table 3:** Genetic diversity of local or breed populations

Local or breed population	MNA±SD	N_e_±SD	H_o_±SD	H_e_±SD	*F*
MH	4.46±1.33	3.34±1.32	0.721±0.250	0.724±0.119	0
SCM	8.23±2.77	4.50±1.71	0.638±0.181	0.754±0.107	0.139
NCM	6.15±1.71	3.90±1.26	0.726±0.225	0.731±0.115	0
CR	6.15±2.03	3.99±1.49	0.638±0.229	0.746±0.122	0.105
UT	7.84±2.49	5.04±2.20	0.721±0.164	0.792±0.079	0.066
TWB	6.15±1.28	4.28±1.29	0.671±0.214	0.782±0.081	0.100
DR	5.53±2.46	3.23±1.59	0.627±0.217	0.641±0.175	0
PT	4.50±1.83	3.03±1.41	0.574±0.225	0.630±0.186	0.017
LW	4.26±1.11	2.84±0.98	0.589±0.181	0.632±0.170	0.047
DXN	4.07±1.87	3.24±1.35	0.853±0.256	0.685±0.121	0
PXN	4.96±1.79	3.46±1.12	0.712±0.240	0.710±0.131	0

MNA, mean number of alleles per locus; N_e_, effective number of alleles per locus; H_o_ and H_e_, the observed and unbiased expected heterozygosity; F, heterozygote deficiency or inbreeding coefficient.

MH, Mae Hongson; SCM, Southern part of Chiang Mai; NCM, Northern part of Chiang Mai; CR, Chiang Rai; UT, Uttaradit; TWB, Thai wild boars; DR, Duroc; PT, Pietrain; LW, Large White; DXN, Duroc×native crossbred pig; PXN, Pietrain×native crossbred pig.

**Table 4:** The Nei’s (1972) standard genetic distance (below diagonal) and mean F_ST_ estimates (above diagonal) among each pair of 17 populations

Population	MH	SCM	NCM	CR	UT	TWB	DR	LW	PT	DXN	PXN	JQH	LC	MZ	YJ	TI	RC
MH	-	0.075	0.091	0.089	0.079	0.126	0.147	0.158	0.147	0.108	0.122	0.137	0.179	0.156	0.142	0.101	0.113
SCM	0.287	-	0.054	0.037	0.039	0.081	0.120	0.130	0.118	0.090	0.080	0.142	0.111	0.122	0.108	0.065	0.075
NCM	0.354	0.261	-	0.068	0.055	0.101	0.091	0.122	0.097	0.086	0.084	0.127	0.155	0.126	0.129	0.092	0.098
CR	0.312	0.127	0.300	-	0.046	0.085	0.107	0.118	0.103	0.095	0.086	0.160	0.129	0.127	0.122	0.086	0.093
UT	0.328	0.177	0.277	0.184	-	0.063	0.088	0.103	0.086	0.074	0.069	0.128	0.114	0.107	0.093	0.062	0.075
TWB	0.606	0.454	0.554	0.423	0.352	-	0.127	0.150	0.126	0.113	0.107	0.166	0.138	0.143	0.132	0.082	0.102
DR	0.558	0.551	0.354	0.418	0.371	0.579	-	0.082	0.064	0.095	0.111	0.199	0.199	0.168	0.183	0.131	0.149
LW	0.535	0.520	0.433	0.399	0.380	0.619	0.233	-	0.067	0.139	0.112	0.221	0.235	0.178	0.190	0.152	0.165
PT	0.544	0.526	0.377	0.388	0.356	0.567	0.185	0.160	-	0.097	0.113	0.208	0.209	0.149	0.181	0.130	0.146
DXN	0.370	0.406	0.348	0.383	0.319	0.526	0.317	0.448	0.318	-	0.114	0.155	0.182	0.145	0.153	0.114	0.130
PXN	0.474	0.369	0.358	0.353	0.305	0.520	0.407	0.335	0.408	0.450	-	0.182	0.166	0.138	0.148	0.103	0.118
JQH	0.459	0.588	0.474	0.629	0.532	0.702	0.712	0.719	0.735	0.534	0.686	-	0.222	0.190	0.187	0.138	0.147
LC	0.615	0.420	0.619	0.468	0.452	0.542	0.702	0.771	0.742	0.663	0.604	0.741	-	0.216	0.171	0.114	0.134
MZ	0.590	0.544	0.526	0.518	0.486	0.659	0.627	0.595	0.533	0.540	0.521	0.651	0.764	-	0.158	0.125	0.146
YJ	0.523	0.471	0.559	0.492	0.416	0.611	0.702	0.657	0.691	0.592	0.607	0.659	0.584	0.566	-	0.101	0.111
TI	0.462	0.350	0.524	0.456	0.369	0.484	0.646	0.665	0.628	0.567	0.519	0.579	0.431	0.582	0.442	-	0.055
RC	0.456	0.359	0.480	0.421	0.392	0.543	0.648	0.643	0.631	0.581	0.534	0.557	0.489	0.618	0.435	0.216	-

MH, Mae Hongson; SCM, Southern part of Chiang Mai; NCM, Northern part of Chiang Mai; CR, Chiang Rai; UT, Uttaradit; TWB, Thai wild boars; DR, Duroc; LW, Large White; PT, Pietrain; DXN, Duroc×native crossbred pig; PXN, Pietrain×native crossbred pig; JQH, Jiangquhai; LC, Luchuan; MZ, Min; YJ, Yushanhei; TI, Tibetan; RC, Rongshang.
