# Supplementary material for: Genetic diversity analysis of Thai indigenous pig population using microsatellite markers
Source: Asian-Australas J Anim Sci. 2019 Mar 7;32(10):1491–500. doi: 10.5713/ajas.18.0832 (PMC6718910; doi:10.5713/ajas.18.0832)
Supplement: Supplementary file 1 [file ajas-18-0832-suppl.pdf]

SUPPLEMENTARY INFORMATION

Supplementary Table S1. Porcine microsatellite markers with chromosome location (SSC), annealing temperature (TA), expected fragment size (EFS), and observed fragment size (OFS)

Marker	SSC	TA (ºC)	EFS (bp)	OFS (bp)
S0155 *	1	53	150-166	141-165
CGA *	1	57	266-302	214-320
S0226 *	2	57	181-205	175-216
SW240 *	2	57	90-115	88-120
S0002 *	3	61	190-216	184-254
SW72 *	3	61	90-120	94-112
S0227 *	4	61	231-256	225-265
IGF-1 *	5	56	197-209	189-207
S0005 *	5	57	205-248	201-247
SW122 *	6	57	110-122	99-133
S0101 *	7	56	197-216	195-223
SW632 *	7	57	159-180	144-176
S0225 *	8	52	170-196	167-193
SW911 *	9	61	153-177	144-172
SW951 *	10	59	120-136	120-132
S0386 *	11	50	156-172	150-190
S0090 *	12	58	244-251	229-249
S0068 *	13	64	211-260	207-257
S0215 *	13	64	135-169	123-183
SW857 *	14	61	144-160	138-164
S0355 *	15	56	243-277	241-269
SW936 *	15	57	80-117	75-119
S0026 *	16	59	92-106	85-111
SW1031	17	57	93-107	87-116
S0120	18	57	154-176	148-181
S0218 *	X	61	166-204	156-188

* ISAG/ FAO, 2004.
